# Modulation of Corticotropin-Releasing Hormone Receptor Expression During In Vitro Keratinocyte Differentiation

**DOI:** 10.3390/cimb48020210

**Published:** 2026-02-14

**Authors:** Carole-Anne Martins, Sara Lesink, Angéline Roux, Guillaume Collet, Richard Daniellou

**Affiliations:** 1 Cosmetology, AgroParisTech, 45100 Orléans, France; 2 Umaï, 111, Boulevard Duhamel du Monceau, 45166 Olivet, France; sara@umai-natural.com (S.L.); angeline@umai-natural.com (A.R.); 3 INRAE, AgroParisTech, UMR Micalis, Université Paris-Saclay, 78350 Jouy-en-Josas, France

**Keywords:** keratinocyte differentiation, corticotropin-releasing hormone receptors (CRHR1/2), calcium-induced differentiation, modulation of expression, skin

## Abstract

Corticotropin-releasing hormone (CRH) and its receptors CRHR1 and CRHR2 are major actors in the stress response and are well established as components of the hypothalamic–pituitary–adrenal (HPA) axis. Evidence also suggests they are expressed in peripheral tissues and, more interestingly, in the skin. While CRHR1 expression in keratinocytes is documented in terms of presence or absence, data on CRHR2 remain sparse. Moreover, there is no detailed description of the exact localization of CRHR1/2 receptors within the different layers of the epidermis, leaving this question fully unexplored. To better understand the link between stress and skin disorders, we aimed to investigate the differential expression of CRHR1 and CRHR2 in keratinocytes, depending on their level of differentiation. In vitro results demonstrated that CRHR1 appears to be more abundant at early stages of differentiation and CRHR2 at more advanced stages.

## 1. Introduction

Stress is now well recognized as a major global health concern [1], as demonstrated by surveys conducted in 77 countries indicating that psychological stress is experienced by 30–50% of the population [2]. Chronic stress is increasingly associated with the aggravation of various diseases, including cardiovascular disease [3,4] and skin diseases, such as atopic dermatitis and psoriasis, or skin tumors, largely due to the increased inflammatory response [5,6]. Among the biological mechanisms activated during stress, the hypothalamic–pituitary–adrenal (HPA) axis, and particularly the cortisol pathway, is one of the best-characterized. Indeed, glucocorticoids are well known to maintain immune homeostasis and regulate inflammation [7,8].

**Scheme 1:**
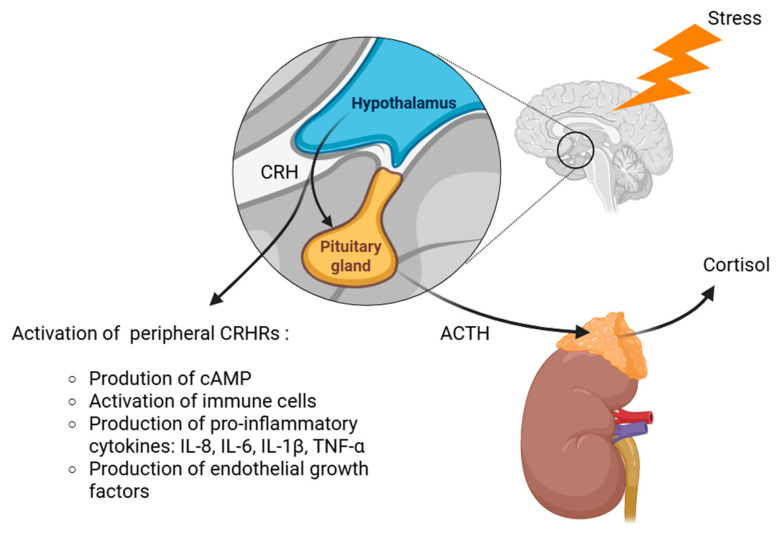
Cortisol pathway and the role of CRHRs. *Created in BioRender. Martins, C. (2026) https://BioRender.com/yqp8kis.*

Corticotropin-releasing hormone (CRH) is one of the molecules of the cortisol pathway produced by the HPA axis during a period of stress (Scheme 1). CRH binds two main types of membrane receptor belonging to the G protein-coupled receptor (GPCR) family: CRHR1 and CRHR2. CRHR1, which shows a high affinity for CRH, is predominantly expressed in the hypothalamus. Its activation stimulates the secretion of adrenocorticotropic hormone, leading to the release of cortisol from the adrenal cortex [9]. In contrast, CRHR2 displays a more peripheral tissue distribution, with high expression in the myocardium, blood vessels, and other peripheral tissues. Although CRHR2 has a lower affinity for CRH compared to CRHR1, it plays a key role in cardiovascular regulation, sympathetic nervous system modulation, and certain metabolic and immune functions [9].

Cortisol is well known to regulate inflammation [7,8], but under conditions of acute or chronic stress, this regulatory system can become dysregulated. Excessive or prolonged activation of the HPA axis exacerbates inflammation [10]. One contributing factor is the direct pro-inflammatory action of CRH. When receptors are activated, they not only stimulate adrenocorticotropic hormone (ACTH) or cyclic adenosine monophosphate (cAMP) (Scheme 1) [11] but also promote the production of pro-inflammatory cytokines such as interleukin 6 (IL-6), tumor necrosis factor α (TNF-α), and interleukin 1β (IL-1β) for CRHR1 [12,13,14,15], and interleukin 8 (IL-8) for CRHR2 [16,17] (Scheme 1).

Interestingly, CRHR1 has also been identified in peripheral tissues, particularly in the skin, where it is expressed by keratinocytes, melanocytes, and fibroblasts [18,19,20,21]. In contrast, the expression of CRHR2 in the skin is still poorly documented. While studies have reported its presence in follicular keratinocytes, others suggest its absence in interfollicular epidermal keratinocytes [18,21]. Although the roles of CRH receptors have been extensively studied in the brain, their functions in the skin remain poorly understood. This extraneural localization is particularly intriguing, given the well-established link between psychological stress and the disruption of skin barrier function, such as that found during atopic dermatitis or psoriasis. It suggests a potential involvement of these CRH receptors in cutaneous stress responses.

The skin’s barrier function is provided by the epidermis and its predominant cell type: keratinocytes. Whereas CRHR1 expression is well established in skin, CRHR2 expression remains controversial. Moreover, nothing is known about whether their expression depends on keratinocyte differentiation. Therefore, a better understanding of their differential expression and precise localization within the skin would facilitate the study of their mechanisms of action and their potential link to stress-linked pathologies.

In the present study, we have investigated the expression patterns of CRHR1 and CRHR2 receptors in keratinocytes in vitro at different levels of differentiation. The first part of our work was carried out in vitro with the use of the common HaCaT cell line obtained from human immortalized keratinocytes [22,23,24]. In vitro differentiation of HaCaT cells was induced using the well-established modulation of calcium gradient [25,26,27]. The differentiation status was verified using two relevant markers: cytokeratin 10 (K10), an early marker of differentiation expressed in the spinous layer of skin [28], and involucrin, a marker for which the expression progressively increases from the spinous to the granular layer of skin [29,30]. Once differentiation was established and confirmed, CRHR1/2 levels of expression were qualitatively studied using immunocytochemistry (ICC) and quantitatively studied using Western blot, both at various calcium concentrations. In the second part of the study, the results obtained from HaCaT cell culture were compared with immuno-labelling of CRHR1/2 within an advanced model of skin tissue. Reconstructed skins are made with normal human epidermal keratinocyte (NHEK) and normal human dermal fibroblast (NHDF), allowing one to reproduce in vitro an epidermis and a dermis [31]. Built to mimic skin behavior and structure, the reconstructed skin provides keratinocytes at all levels of differentiation, from the germinative layer up to corneocytes.

## 2. Materials and Methods

### 2.1. Cell Culture

HaCaT cells were purchased from the cell provider CLS (300493, Cytion GmbH, Heidelberg, Germany). Cells were maintained in Dulbecco’s Modified Eagle Medium (DMEM, 42430025, Gibco, Grand Island, NY, USA) supplemented with 10% fetal bovine serum (FBS, 10270-106, Gibco, Grand Island, NY, USA), penicillin 100 U/mL, and streptomycin 100 μg/mL (P4333, Sigma-Aldrich, Saint-Louis, MO, USA). Cells were cultured under sterile conditions in 10 cm Petri dishes at 37 °C in a humidity-saturated incubator with 5% of CO_2_. Cells were detached with 0.25% trypsin-EDTA (25200-056, Gibco, Grand Island, NY, USA) and seeded in new Petri dishes.

### 2.2. Calcium-Mediated Differentiation

All experiments related to cell differentiation were carried out in supplemented calcium-free medium in which a well-defined concentration of Ca^2+^ was added to obtain cells at different levels of differentiation. This protocol was inspired by the well-established calcium-induced method of in vitro keratinocyte differentiation [23,26,27,32]. Briefly, FBS was treated with 5% (*w*/*w*) Chelex 100 (C7901, Sigma-Aldrich, Saint-Louis, MO, USA), following the manufacturer’s protocol, to eliminate FBS with Ca^2+^. Then, Dulbecco’s Modified Eagle’s Medium without calcium (DMEM, 21036029, Gibco, Grand Island, NY, USA) was supplemented with the 10% calcium-free FBS (*v*/*v*), penicillin (100 U/mL), streptomycin (100 μg/mL), and L-glutamine (4 mM) (35050-061, Thermo Fisher Scientific, Waltham, MA, USA). Four final Ca^2+^ concentrations were investigated, 0 mM, 0.1 mM, 1.2 mM, and 1.8 mM, all prepared from a 1 M calcium chloride stock solution (21115, Sigma-Aldrich, Saint-Louis, MO, USA). All resulting cell culture media were verified using a colorimetric calcium assay according to the manufacturer’s instructions (E-BC-K103-M, Elabscience, Houston, TX, USA). Prior to differentiation induction, HaCaT cells were cultured for at least two weeks in calcium-free medium (0 mM) to restore a basal and undifferentiated state. Subsequently, the 4 various calcium concentration gradients were applied for 7 h, 24 h, and 48 h to induce cell differentiation.

### 2.3. Immunocytochemistry (ICC)

Immunocytochemistry (ICC) was performed on HaCaT cells seeded in black flat-bottom 96-well plates (3631, Corning, New York, NY, USA). Cells were seeded at 5000 cells/well and were cultivated until reaching 70% confluency. After fixation with 4% (*w*/*v*) paraformaldehyde in PBS (J19943-K2, Thermo Fisher Scientific, Waltham, MA, USA), permeabilization with 0.5% (*v*/*v*) Triton X-100 in PBS (T8787, Sigma-Aldrich, Saint-Louis, MO, USA), and treatment with 20 mM urea (GE17-1319-01, Sigma-Aldrich, Saint-Louis, MO, USA) in PBS, the plates were blocked overnight at 4 °C with PBS (P4474, Sigma-Aldrich, Saint-Louis, MO, USA) containing 1% bovine serum albumin (BSA) (DY995, R&D Systems, Minneapolis, MN, USA). Cells were then incubated with rabbit anti-hCRHR1 (ACR-050-200UL, Alomone Labs, Jerusalem, Israel) or anti-hCRHR2 (ACR-052-200UL, Alomone Labs, Jerusalem, Israel) polyclonal IgG antibodies both used at 10 µg/mL in 1% (*w*/*v*) PBS-BSA and incubated for 2 h at RT. After five washes with 100 µL of PBS, cells were incubated with an Alexa Fluor-488 conjugated goat anti-rabbit IgG secondary antibody (A-1108, Invitrogen, Carlsbad, CA, USA) at 10 µg/mL in 1% PBS-BSA for 1.5 h at RT. After five washes with 100 µL of PBS, nuclei were counterstained with DAPI (R37606, NucBlue Fixed Cells, Invitrogen, Carlsbad, CA, USA). Several controls were realized, including the use of a rabbit IgG Isotype Control (31235, Invitrogen, Carlsbad, CA, USA) and the secondary antibody control alone (A-1108, Invitrogen, Carlsbad, CA, USA). These two antibodies were incubated in the same conditions and concentrations previously mentioned. Pictures were obtained with a Leica DMi8 inverted fluorescent microscope (Leica Microsystems, Wetzlar, Germany) equipped with a Leica K3C charge-coupled device (CCD) and color camera. DAPI fluorescence was detected with a filter cube composed of a 405 ± 60 nm excitation filter, a 455 nm beam splitter, and a 470 ± 40 nm emission filter. Alexa Fluor-488 fluorescent signal was detected with a filter cube composed of a 470 ± 40 nm excitation filter, a 495 nm beam splitter, and a 525 ± 50 emission filter. All acquisitions were performed with a 20× objective. High-resolution mosaic images were created from multiple images with the LAS-X Office software (Version 3.10.0.28982).

### 2.4. Western Blot

After the incubation with the calcium concentration gradient, 10^6^ cells were mechanically lysed in 2 mL of PBS by repeated aspiration through a sterile syringe with a 0.45 mm diameter needle (26 gauge). Protein concentrations of cell lysate were determined using the BCA Protein Assay Kit (71285-M, Sigma-Aldrich, Saint-Louis, MO, USA). Samples for the Western blot were prepared by mixing equal volumes of protein lysate with 2× concentrated Laemmli buffer (1610737, Bio-Rad, Hercules, CA, USA) containing 0.5% (*w*/*w*) dithiothreitol (DTT, 1610610, Bio-Rad, Hercules, CA, USA) in PBS. After mixing, samples were heated at 90 °C for 5 min at 300 rpm (Thermomixer C, Eppendorf, Hambourg, Germany). Gels were loaded with 10 µg of proteins per well. A Western C molecular weight marker (1610376, Bio-Rad, Hercules, CA, USA) was loaded in an outer lane as a size marker. Proteins were separated by SDS-PAGE electrophoresis using 4–20% precast stain-free polyacrylamide gels (4568094, Bio-Rad, Hercules, CA, USA). Electrophoresis was performed first at 50 V for 5 min followed by 150 V for 35 min. After migration, the gel was activated for 5 min under UV light (emission filter: 590/110 nm, Chemidoc^®^ Trans illumination mode, Biorad, Hercules, CA, USA). After activation, proteins were transferred onto a nitrocellulose membrane using a semi-dry transfer system (Trans-Blot Turbo™, Biorad, Hercules, CA, USA) at 2.5 A constant for 5 min. The membrane was then blocked overnight at 4 °C with 10 mL of EveryBlot blocking buffer (12010020, Bio-Rad, Hercules, CA, USA). Membranes were incubated with a rabbit anti-hCRHR1 (ACR-050-200UL, Alomone Labs, Jerusalem, Israel) or hCRHR2 (ACR-052-200UL, Alomone Labs, Jerusalem, Israel) polyclonal IgG antibodies diluted at 4 µg/mL in Everyblot blocking buffer for 2 h at RT. Cytokeratin K10 was detected with rabbit anti-K10 (ACR-310249, Sigma-Aldrich, Saint-Louis, MO, USA) polyclonal IgG antibodies diluted at 2 µg/mL in Everyblot blocking buffer for 2 h at RT. Involucrin was detected by using mouse monoclonal IgG1 primary antibody against human involucrin (I9018, Sigma-Aldrich, Saint-Louis, MO, USA) diluted at 0.67 µg/mL in Everyblot blocking buffer for 2 h at RT. After incubation with primary antibodies, membranes were washed five times with 10 mL of tris buffer saline (TBS, J60764.K2, Thermo Fisher Scientific, Waltham, MA, USA) containing 0.1% (*v*/*v*) Tween 20 (1610781, Bio-Rad, Hercules, CA, USA). Then, membranes incubated with a rabbit primary antibody were incubated with 0.2 µg/mL of goat HRP-conjugated anti-rabbit secondary antibody and 2 µL of streptactin-HRP in 10 mL of EveryBlot blocking buffer. Membranes incubated with a mouse primary antibody were incubated with 2 µg/mL of HRP-conjugated anti-mouse secondary antibody and 2 µL of streptactin-HRP in 10 mL of EveryBlot blocking buffer. All secondary antibodies were incubated for 2 h at RT in the dark. Prior to imaging, the membranes were washed five times with 10 mL of TBS containing 0.1% Tween 20. Detection was performed using Clarity Western ECL substrate (1705061, Bio-Rad, Hercules, CA, USA) for 5 min, and the membrane was imaged on a ChemiDoc system. The amount of protein per lane for stain-free images was assessed using the “stain-free gel” mode in the ChemiDoc system (UV trans-Illumination with a 590/110 emission filter) and quantified using the “lane” volume function in Image Lab v6.0 (Bio-Rad, Hercules, CA, USA). The total amount of protein per lane was normalized to the lane representing 7 h of incubation in the 0 mM calcium condition. The normalization factor was used to readjust differences, if any, in protein loading for all lanes. Various proteins of interest were detected with “Chemiluminescence” mode (no excitation, shortpass 647 nm emission filter). Data are presented as the fold change from the band intensity of the 7 h incubation at 0 mM calcium. Statistical analysis between the different cell incubation conditions was performed using Student’s *t*-test, with *p*-values adjusted by the Bonferroni correction. Analyses were carried out using RStudio software (Version 2025.05.0).

### 2.5. Immunohistochemistry (IHC)

The reconstructed skins (T-Skin™) were provided by Episkin company (Lyon, France). They were assembled using human fibroblasts and keratinocytes, both from a 35-year-old Caucasian female donor. T-Skin samples were fixed with 4% (*w*/*v*) paraformaldehyde in PBS prior to being dehydrated and embedded in paraffin. Sections of 7 µm thickness were prepared from T-Skin™ containing paraffin blocks using a HistoCore BIOCUT microtome (Leica Microsystems, Wetzlar, Germany). The sections were collected on Superfrost Plus ™ microscope slides (10149870, Epredia™, Neuilly-sur-seine, France), dried on a heating plate at 40 °C and incubated overnight at 37 °C. Slides were deparaffinized and rehydrated to water, first with xylen, then with alcohols from 100% to 50%, and lastly with pure water. Antigen retrieval was performed by heating the slides in a 0.1 M citrate buffer, pH 6 (J62918, Thermo Fisher scientific, Waltham, MA, USA), at 90 °C for 30 min in a water bath. The slides were then rinsed under cold tap water for 10 min. Rinsed slides were then washed twice with TBS containing 0.1% (*v*/*v*) Tween-20 (TBST). Then, slides were incubated with 3% (*v*/*v*) hydrogen peroxide in water (H1009, Sigma-Aldrich, Saint-Louis, MO, USA) for 10 min at RT to block endogenous peroxidase activity. A neutralization step was then performed by incubating the slides with 20 mM urea in PBS for 20 min, followed by a TBST wash. Non-specific binding was blocked by incubating the slides in TBS with 10% (*w*/*v*) BSA solution for 1 h at RT. After three washes in TBST, the labelling area was delimited on each slide using a hydrophobic barrier pen (22309, TED PELLA, Redding, CA, USA). Immunolabelling was performed with specific antibodies: rabbit anti-hCRHR1 (ACR-050-200UL, Alomone Labs, Jerusalem, Israel) or anti-hCRHR2 (ACR-052-200UL, Alomone Labs, Jerusalem, Israel). Polyclonal IgG antibodies were diluted to a final concentration of 20 µg/mL, and 200 µL of the antibody solution was applied to each delimited section and incubated for 1 h at RT. Slides were then washed three times in TBST. The HRP-conjugated goat anti-rabbit secondary antibody was prepared at a concentration of 10 µg/mL, and 200 µL was applied to each section for 30 min at RT. The substrate was reconstituted using 1 mL of 3,3′-diaminodbenzidine (DAB) solution and 9 mL of peroxide substrate buffer (34002, Thermo Fisher scientific, Waltham, MA, USA). After three additional TBST washes, slides were incubated for 3 min with the substrate solution. Then, they were rinsed three times in TBS and counterstained with Mayer’s hematoxylin (GHS116, Sigma-Aldrich, Saint-Louis, MO, USA) for 5 min, followed by two washes in distilled water. Finally, the slides were dehydrated in absolute ethanol and mounted using Permount Mounting Media (SP15-100, Thermo Fisher Scientific, Waltham, MA, USA). Images were obtained with a Leica DMi8 inverted microscope equipped with a Leica K3C charge-coupled device and color camera. All acquisitions were performed with a 20× objective and processed with LAS-X Office software (Version 3.10.0.28982).

### 2.6. Statistical Analysis

With four groups to analyse, a total of six pairwise comparisons were performed using a Student *t*-test. Each Student *t*-test is associated with a type-I error risk, which is the probability of rejecting the H0 hypothesis when it is true (false positive). Here, the type I error was set at 5% (α = 0.05), in accordance with generally accepted rules. The multiplication of tests increases the probability of obtaining at least one misleadingly significant result. The Bonferroni correction allows the overall risk of a type I error to be controlled by dividing the significance threshold α by the number of comparisons performed. This more conservative approach limits the risks associated with small sample sizes.

## 3. Results

### 3.1. Fluorescence Intensity Assessment of CRHR1 and CRHR2 Expression in HaCaT Cell Line Under Calcium-Induced Differentiation

HaCaT cell differentiation was achieved with incubation at four specific calcium concentrations: 0, 0.1, 1.2, and 1.8 mM. Interestingly, morphological changes were observed after 48 h of incubation with the various calcium concentrations (Figure S1). Indeed, at low calcium concentrations (0 and 0.1 mM), cells appeared smaller and more elongated, whereas at high concentrations (1.2 mM and 1.8 mM), cells became large, flattened, and polygonal. These cell morphological changes are typically associated with differentiated HaCaT cells [24,25], which constitute the first evidence of the calcium-induced modulation of differentiation

To validate the calcium-induced differentiation of keratinocytes, the expression of involucrin (marker of differentiated keratinocytes [29,30]) was assessed. As shown in Figure S2, involucrin expression levels increase significantly between 0 mM and 1.2 mM of calcium (4.27+/−0.32). However, a weak expression was detected at 0 mM calcium concentration. According to our results and together with previously published reports, this condition corresponds to a basal level of expression and to an undifferentiated state of keratinocytes [28]. When the calcium concentration increased from 0 to 0.1 mM, a twofold increase in involucrin fluorescence intensity was observed (2.52+/−0.30). This change corresponds to the “calcium switch,” a well-described threshold largely described in the literature [26,28]. Above this threshold, keratinocytes, including HaCaT cells, begin to differentiate. Therefore, the 0.1 mM condition represents an early stage of differentiation. No significant difference is observed between 1.2 mM and 1.8 mM, which is consistent with already reported data indicating that 1.2 mM is sufficient to induce maximal differentiation [29] (Figure S2). The morphological differences observed, together with the significant increase in involucrin expression, confirm the successfully achieved calcium-induced differentiation of HaCaT cells (Figures S1 and S2).

**Figure 1:**
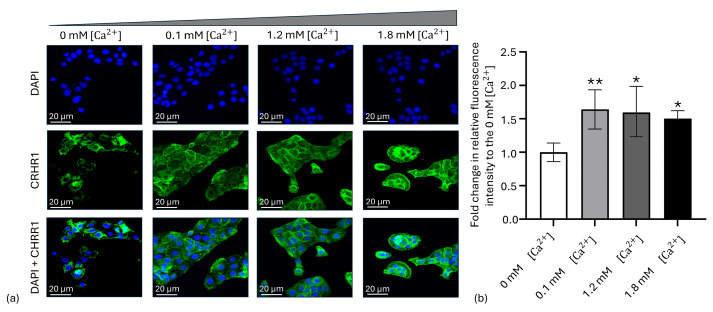
**Figure 1.** Fluorescent microscopy imaging of CRHR1 receptor during in vitro differentiation. (**a**) HaCaT cells were incubated for 48 h in the presence of a calcium gradient. CRHR1 protein was immuno-labelled and detected using an Alexa Fluor 488 fluorophore (green). Nuclei were counterstained with DAPI (blue). Images were acquired with a 200× magnification. Representative pictures. (**b**) Fluorescence intensity measurements of labelled HaCaT cells with a plate reader. Scale bars represent 20 µm. *n* = 3. Bonferroni-adjusted Student *t*-test compared to 0 mM Ca^2+^ concentration with * *p* < 0.05, ** *p* < 0.01.

Once differentiation conditions were established and confirmed, specific antibodies directed against hCRHR1 or hCRHR2 were used for immunolabelling of HaCaT after incubations at various calcium concentrations. Figure 1a shows the results of immunofluorescent labelling for CRHR1 (Figure 1 and Figure S3). At 0 mM calcium, only a few cells or parts of cells were labelled, suggesting low or non-uniform CRHR1 expression when cells are undifferentiated. In contrast, starting from 0.1 mM calcium, most of the cells were positively labelled with a more homogeneous expression of CRHR1. The corresponding fluorescence intensities were quantified and are presented in Figure 1b. Results are expressed as fold change relative to the 0 mM calcium concentration. CRHR1 expression at the 0.1 mM calcium concentration is increased by 1.5 times (+/−0.38) compared to that at 0 mM. As previously mentioned, this concentration corresponds to the “calcium switch” [26,28]. However, no significant differences are found between the 0.1 mM, 1.2 mM, and 1.8 mM calcium concentrations. Thus, CRHR1 expression appears to be low in undifferentiated keratinocytes. Higher expression is observed when the calcium switch is reached. Moreover, this higher-level expression is stable when the calcium concentrations exceed the calcium switch, corresponding to more advanced stages of differentiation.

**Figure 2:**
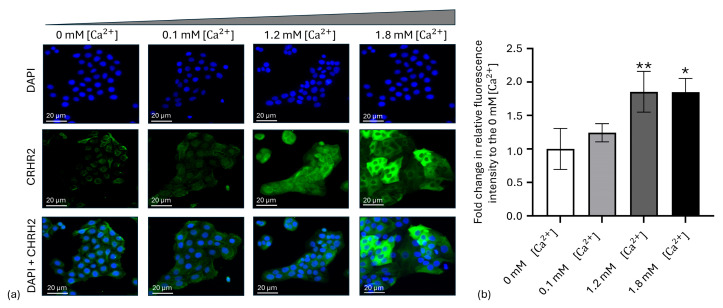
Fluorescent microscopy imaging of CRHR2 receptor during in vitro differentiation. (**a**) HaCaT cells were incubated for 48 h in the presence of a calcium gradient. CRHR2 protein was immuno-labelled and detected using an Alexa Fluor 488 fluorophore (green). Nuclei were counterstained with DAPI (blue). Images were acquired with a 200× magnification. Representative pictures. (**b**) Fluorescence intensity measurements of labelled HaCaT cells with a plate reader. Scale bars represent 20 µm. *n* = 3. Bonferroni-adjusted Student *t*-test compared to 0 mM Ca^2+^ concentration with * *p* < 0.05, ** *p* < 0.01.

Figure 2a shows the results of immunofluorescent labelling for CRHR2 (Figure S3). Weak signals of CRHR2 are detected at 0 or 0.1 mM calcium concentrations, which presumably correspond to a basal level of expression. Higher fluorescence signal intensities are observed at 1.2 and 1.8 mM when compared to the lower calcium concentrations. To confirm these qualitative observations, CRHR2 fluorescence intensities were quantified using a plate reader. As shown in Figure 2b, the signals at 1.2 and 1.8 mM calcium concentrations are approximately twice as high as that observed at the 0 mM concentration (respectively, 1.84+/0.31 and 1.80+/−0.22). Moreover, no significant differences are found between 1.2 mM and 1.8 mM, suggesting that maximum expression is reached at the 1.2 mM calcium concentration. These findings suggest that CRHR2 is gradually expressed when increasing the calcium concentration and, consequently, the level of differentiation. This is particularly noteworthy as CRHR2 has not previously been clearly identified as being expressed in HaCaT cells [18]. Finally, the two receptors, CRHR1 and CRHR2, are found in differentiated keratinocytes, with an increase in expression at calcium concentrations corresponding to the well-described calcium switch and above.

### 3.2. Western Blot Quantification of CRHR1 and CRHR2 Expression in HaCaT Cell Line Under Calcium-Induced Differentiation

Immunocytochemistry results were then consolidated with Western blot experiments conducted for both receptors, CRHR1 and CRHR2. Western blot experiments present some additional information because they provide (i) a higher level of sensitivity, (ii) semi-quantitative results, and (iii) the relative size in Dalton for each of the studied proteins.

**Figure 3:**
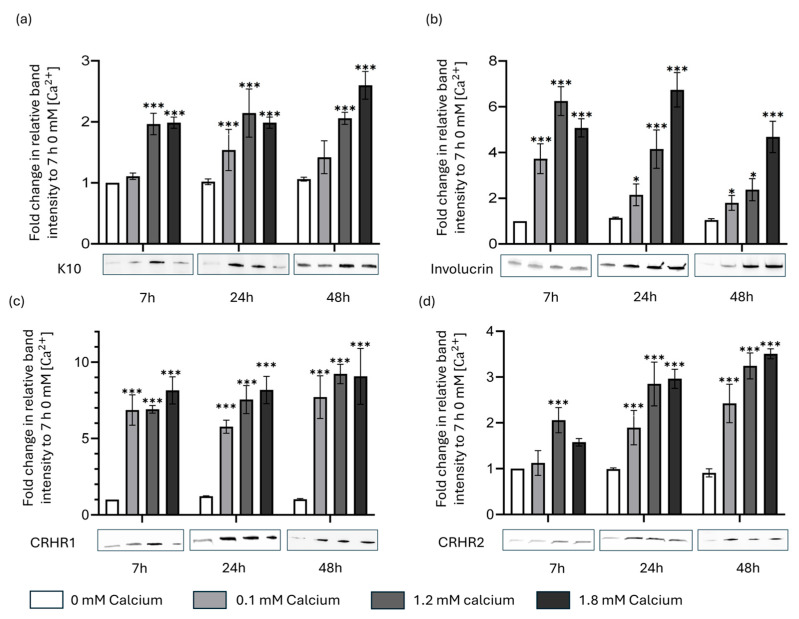
Western blot quantification of CRHR1/R2 expression in HaCaT cells upon calcium-induced differentiation. Protein expression was analysed after 7, 24, and 48 h of incubation at 0, 0.1, 1.2, and 1.8 mM calcium concentrations. Pictures of membranes show representative Western blot bands of (**a**) cytokeratin K10 (Figure S4), (**b**) involucrin (Figure S5), (**c**) CRHR1 (Figure S6), and (**d**) CRHR2 (Figure S7). Graphs display quantifications of band intensities expressed as fold change using the 0 mM calcium concentration at 7 h as a reference value. Values are means ± SD. *n* = 3. Bonferroni-adjusted Student *t*-test compared to 0 mM Ca^2+^ concentration with * *p* < 0.05, *** *p* < 0.001.

First, the expression of two epidermal differentiation markers was investigated to confirm the effectiveness of keratinocyte differentiation conditions. These two markers were cytokeratin K10 (Figure 3a) and involucrin (Figure 3b). Graphs represent the relative expression levels of proteins of interest. Results are expressed as the fold change relative to the reference condition, which is the 7 h incubation with 0 mM calcium.

Involucrin bands appearing around 90 KDa are shown in Figure 3b and are quantified in graphs (Figure 3b and Figure S5). The evolution of involucrin quantities was investigated over time and with changes in calcium concentration. First, significant increases in involucrin expression levels are observed when increasing the calcium concentration, compared to the reference condition (7 h, 0 mM calcium). These results confirm the effective calcium-induced differentiation. The 24 h incubation time emerges as particularly relevant, with fold changes of approximately 2 times at 0.1 mM (+/−0.21), 4 times at 1.2 mM (+/−0.55), and 6.5 times at 1.8 mM (+/−0.53) of calcium concentration.

As a second marker for calcium-induced differentiation, the protein K10 was investigated and is presented in Figure 3a and Figure S4. The expected size of the protein was confirmed with bands appearing around 57 kDa for all incubation times. Higher calcium concentrations, 1.2 mM and 1.8 mM, are associated with a significant cytokeratin K10 overexpression when compared to the 0 mM calcium concentration. Results were more variable at 0.1 mM; only the 24 h and 0.1 mM calcium concentration showed a significant increase relative to 0 mM calcium (1.67+/−0.36). While being less impressive compared to involucrin, the K10 results confirm the effective calcium-induced modulation of expression. Overall, the increase in both differentiation markers, involucrin and cytokeratin K10, confirms the effectiveness of the calcium-induced keratinocyte differentiation in vitro.

With controlled conditions of differentiation established and confirmed, the relative expression of CRHR1 and CRHR2 was investigated.

The CRHR1 bands, with a size around 40 KDa, and their quantifications are presented in Figure 3c. The results confirm the effect of the calcium switch on CRHR1 expression. For all incubation times, a clear increase in CRHR1 levels is observed at the 0.1 mM calcium concentration. After 24 h of incubation, protein expression is approximately 5.5 times higher at 0.1 mM (+/−0.39), 7.5 times at 1.2 mM (+/−1.40), and 8 times at 1.8 mM (+/−1.42) compared to the 0 mM calcium concentration condition. However, no significant differences are observed between 0.1 mM, 1.2 mM, and 1.8 mM, which confirms our previous ICC results (Figure 1). Additionally, no major variation is observed across incubation time (Figure S8), suggesting that CRHR1 expression is rapidly induced and remains stable up to 48 h.

The CRHR2 bands, with a size around 40 KDa, are presented in Figure 3d. Interestingly, the Western blot images and quantifications revealed detectable CRHR2 expression even at 0 mM and 0.1 mM calcium, with visible bands (Figure 3d), which had not been clearly observed with ICC experiments. It clearly confirms the higher level of sensitivity between ICC and Western blot experiments, and it indicates the presence of a basal level of CRHR2 expression with undifferentiated keratinocytes.

Focusing on quantifications, in Figure 3d and Figure S8, CRHR2 expression appears to increase more progressively over time. A significant increase in protein expression was observed for all calcium concentrations between 7 h, 24 h, and 48 h (Figure S8). Although fold changes are lower than for CRHR1, they still increase over time, from 1.1 (+/−0.31) at 7 h to 1.8 (+/−0.30) at 24 h and 2.4 (+/−0.46) at 48 h for the 0.1 mM calcium concentration condition. After 24 h of incubation, CRHR2 expression shows a progressive increase. A significant difference is observed between the calcium-free condition and 0.1 mM calcium, with the fold change rising from 1 (+/−0.07) to 1.8 (+/−0.30). A further significant increase is detected between the low 0.1 mM calcium concentration and the high 1.2 mM and 1.8 mM calcium concentrations, reaching fold changes of 2.8 (+/−0.49) and 2.9 (+/−0.21), respectively. These data confirm that CRHR2 is predominantly expressed at high calcium concentrations, corresponding to more advanced stages of keratinocyte differentiation.

### 3.3. Differential Expression of CRHR1 and CRHR2 in a Reconstructed Skin Model

Reconstructed skin models were used to correlate in vitro observations of the HaCaT cell line upon calcium-induced differentiation with normal primary keratinocytes differentiated, in a layer-per-layer manner, within a reconstructed skin.

**Figure 4:**
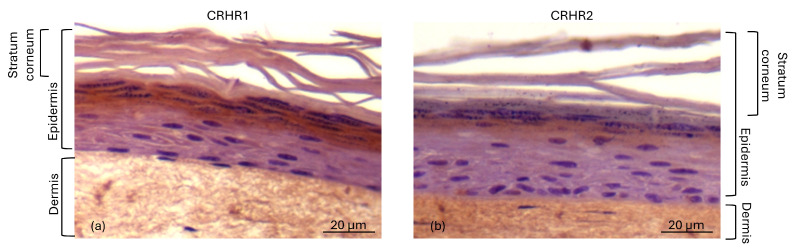
Optical microscopy imaging of CRHR1 and CRHR2 proteins in human reconstructed skin. Tissue sections of reconstructed skin (T-Skin™) were labelled with anti-hCRHR1 antibodies (**a**) and anti-hCRHR2 antibodies (**b**). CRHR1 and CRHR2 signals appear in brown. Counterstaining is obtained with Mayer’s hematoxylin. Optical microscopy images were acquired with a 200× magnification. Representative pictures. Scale bars represent 20 µm.

Pictures of histological sections (IHC) realized from these T-Skin™ samples clearly show the layered organization of cells (Figure 4a,b and Figure S9). This phenomenon of structuration is accompanied by differentiation occurring from the bottom to the upper side. The more differentiated keratinocytes are located toward the upper side of the epidermis, whereas the less differentiated ones are found at the base and correspond to the germinative layer (Figure 4a,b and Figure S9).

CRHR1 labelling (Figure 4a) becomes more intense in the uppermost region of the epidermis, where keratinocytes are at their final stage before differentiating into corneocytes to form the stratum corneum (compactum and disjunctum). However, the stratum disjunctum is clearly not labelled.

While less pronounced than CRHR1 labelling, CRHR2 labelling shows a similar localization, as observed in Figure 4b. This less intense brown coloration demonstrates that the CRHR2 protein is probably less abundant. Additionally, CRHR2 has been previously described only in hair follicle keratinocytes [18]. The herein reported localization represents an additional finding by showing its expression in normal skin keratinocytes, since the reconstructed skin model does not have hair follicles.

Taken together, these results for CRHR1 and CRHR2 within reconstructed skin are highly correlated with the results obtained with HaCaT cell differentiations achieved in vitro by changing the calcium concentration. The more the keratinocytes are differentiated, the more they express both CRHR1 and CRHR2 receptors.

In light of the present results, two interesting conclusions arise from observations: (i) the cross-validation of the HaCaT cell line differentiated in vitro by the use of calcium with the normal human epidermal keratinocyte (NHEK) differentiated in a layer-per-layer manner within the reconstructed skin, and (ii) the confirmation in two different models that the expression of CRHR1 and CRHR2 is correlated with the level of differentiation.

Finally, the dermis is stained light brown, probably due to nonspecific matrix labelling, which is absent in the isotype control (Figure S9). This nonspecific signal may result from antibody trapping within the extracellular matrix or cross-reactivity with dermal components such as collagen or elastin.

## 4. Discussion

As is well described in the literature, keratinocyte differentiation is tightly controlled by calcium concentration [25,28]. This calcium-dependent regulation of differentiation is clearly demonstrated in skin and is fully applicable to cell culture in vitro. The in vitro ability to tune the level of differentiation of keratinocytes was confirmed in our research with HaCaT cells. Indeed, morphological changes were observed (Figure S2) [24,25] together with the increased expression of differentiation markers such as involucrin and cytokeratin K10 (Figure 3) [28,29,32].

With the in vitro calcium-induced differentiation of keratinocyte validated, a deep focus was placed on the expression of both CRHR1 and CRHR2. Investigations were first conducted by immunolabelling living cells, followed by fluorescent microscopy. Qualitative information gained through cell imaging was further confirmed by Western blot quantification to accurately appreciate the variation in CRHR1/2 expression depending on different levels of keratinocyte differentiation. Our studies confirmed through microscopy and Western blot the presence of CRHR1 in the HaCaT cell line, which is in accordance with existing data reported in the literature [18,21]. CRHR2 imaging and quantifications are more controversial in the literature. Indeed, while previous published studies reported its presence only in specific compartments, such as the hair follicle, others mentioned its absence in the HaCaT cell line [18]. Despite the previously reported publications, here, we have clearly demonstrated the presence of CRHR2 in HaCaT cells [18].

Additionally, a change in CRHR1/2 expression is demonstrated that depends on the calcium concentration incubated with HaCaT cells. This change in expression is directly correlated with the differentiation stage of keratinocytes, in that the more they are differentiated toward corneocytes, the more they express both CRHR1 and CRHR2.

Our results show the induction of CRHR1 around the 0.1 mM calcium concentration, corresponding to the “calcium switch” threshold. This observation means that CRHR1 expression is associated with the early stages of keratinocyte differentiation. CRHR2 induction appears at high calcium concentrations (≥1.2 mM), suggesting that CRHR2 is a late differentiation marker that appears in more advanced stages of keratinocyte differentiation. Additionally, these results suggest that the HaCaT cell line is a relevant in vitro model to study both CRHR1 and CRHR2 receptors, including their calcium-induced regulation of expression.

Our in vitro results obtained using HaCaT cells were further confirmed with more advanced reconstructed skin models (T-Skin™). The presence of CRHR1 and CRHR2 is first confirmed inside the epidermis layer. Moreover, their expression clearly increases when approaching the upper layer of the epidermis, corresponding to more differentiated keratinocytes. This observation is directly correlated with in vitro observations. These results highlight the differential regulation of CRHR1 and CRHR2 during the keratinocyte differentiation occurring in structured and organized reconstructed skin. Additionally, this model is assembled with “normal” cells as compared to the in vitro HaCaT cell line.

This observation is particularly relevant since some skin diseases, such as atopic dermatitis and psoriasis, are characterized by impaired keratinocyte differentiation and proliferation, largely due to disruption of the intracellular calcium gradient [33,34,35]. In addition, people exposed to environmental stress, such as UV exposure, or psychological stress have significantly higher levels of CRH in the skin than unstressed people [19,36]. It is therefore likely that the differentiation and proliferation defects observed in these skin disorders influence CRH receptor expression, thereby exacerbating inflammation.

Despite the light that we have shed on CRHR1/2 regulation by keratinocytes, depending on their levels of differentiation, further studies are warranted involving in vivo validation and functional receptor assays. Indeed, the presence of a receptor does not ensure its functionality, and experiments carried out in vitro on cell culture do not reflect the complexity of the skin in vivo.

Nevertheless, our findings enrich the current understanding of cutaneous CRHR mapping and open new avenues for exploring the roles of these receptors in the skin and/or in keratinocyte cultures.

## Data Availability

The original contributions presented in this study are included in the article/Supplementary Material. Further inquiries can be directed to the corresponding authors.

## Supplementary Materials

The following supporting information can be downloaded at https://www.mdpi.com/article/10.3390/cimb48020210/s1.

## Abbreviations

ACTH	adrenocorticotropic hormone
BCA	bicinchoninic acid
BSA	bovine serum albumin
cAMP	cyclic adenosine monophosphate
CCD	charge-coupled device
CRH	corticotropin-releasing hormone
CRHR	corticotropin-releasing hormone receptor
CRHR1/2	corticotropin-releasing hormone receptor 1/2
DAB	3,3′-diaminobenzidine
DAPI	4′,6-diamidino-2-phenylindole
DMEM	Dulbecco’s modified Eagle’s medium
DTT	dithiothreitol
ECL	enhanced chemiluminescence
EDTA	ethylenediaminetetraacetic acid
FBS	fetal bovine serum
GPCR	G protein-coupled receptor
HPA	hypothalamic–pituitary–adrenal
HRP	horse radish peroxidase
ICC	immunocytochemistry
IgG	immunoglobulin G
IHC	immunohistochemistry
IL-1β	interleukin 1β
IL-6	interleukin 6
IL-8	interleukin 8
NHDF	normal human dermal fibroblast
NHEK	normal human epidermal keratinocyte
TBS	Tris-buffered saline
TBST	Tris-buffered saline with Tween 20
TNF-α	tumor necrosis factor α
PAGE	polyacrylamide gel electrophoresis
PBS	phosphate-buffered saline
SDS	sodium dodecyl sulfate
UV	ultraviolet
2D	two-dimensional
3D	three-dimensional
