# Supplementary material for: Modulation of Corticotropin-Releasing Hormone Receptor Expression During In Vitro Keratinocyte Differentiation
Source: Curr Issues Mol Biol. 2026 Feb 14;48(2):210. doi: 10.3390/cimb48020210 (PMC12939058; doi:10.3390/cimb48020210)
Supplement: Supplementary file 1 [file cimb-48-00210-s001.zip › Supporting Information.pdf]

Supporting Information for:

Modulation of Corticotropin-Releasing Hormone Receptors Expression During Keratinocytes Differentiation

Carole-Anne Martins [1,2], Sara Lesink [2] , Angéline Roux [2] , Guillaume Collet [1*] and Richard Daniellou [1,3*]

[1] Chair of Cosmetology, AgroParisTech, 45100 Orléans, France;
[2] Umaï, 11, boulevard Duhamel du Monceau, 45166 Olivet, France;
[3] INRAE, AgroParisTech, UMR Micalis, Université Paris-Saclay, 78350 Jouy-en-Josas, France
* Correspondings authors: richard.daniellou@agroparistech.fr and guillaume.collet@agroparistech.fr

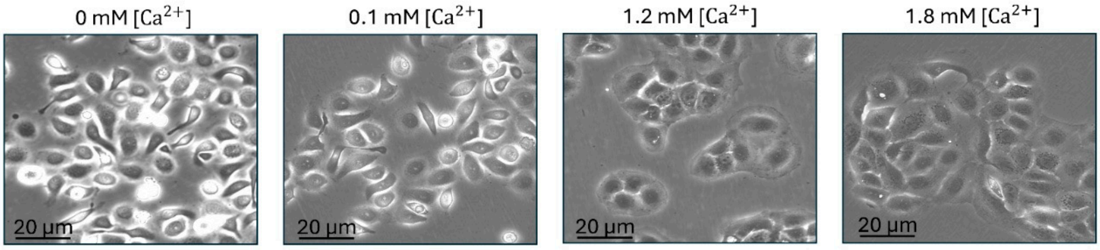

Figure S1: Morphological changes of HaCaT cells in response to the calcium gradient. HaCaT cells were incubate for 48 hours in the presence of various calcium concentrations, respectively 0, 0.1, 1.2 and 1.8 mM. Images were acquired using an optical microscope equipped with a phase contrast at 200X magnification.

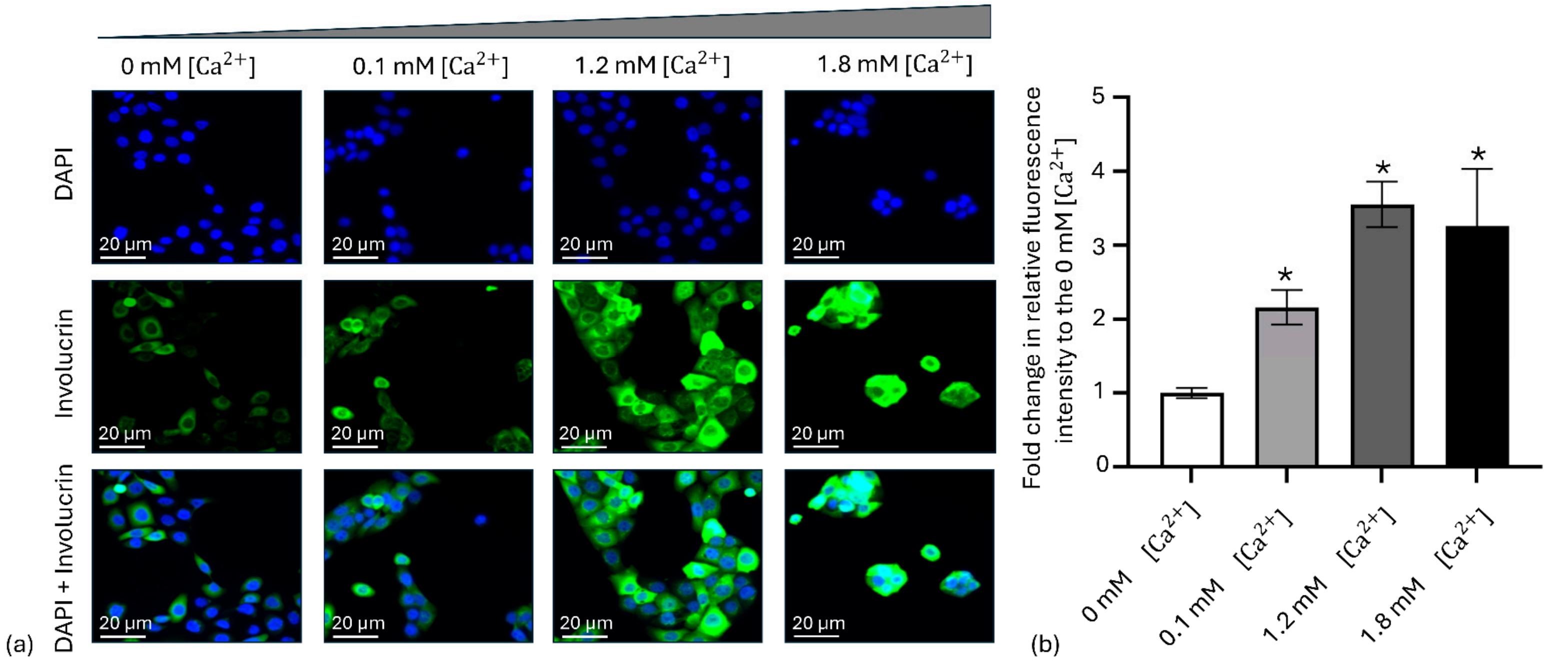

Figure S2: Fluorescent microscopy imaging of involucrin during in vitro differentiation. (a) HaCaT cells were incubated for 48 h in the presence of a calcium gradient. Involucrin protein was immuno-labeled and detected using an Alexa Fluor 488 fluorophore (green). Nuclei were counterstained with DAPI (blue). Images were acquired with a 200x magnification. Representative pictures. (b) Fluorescence intensities measurements of labeled HaCaT cells with a plate reader. n = 3.

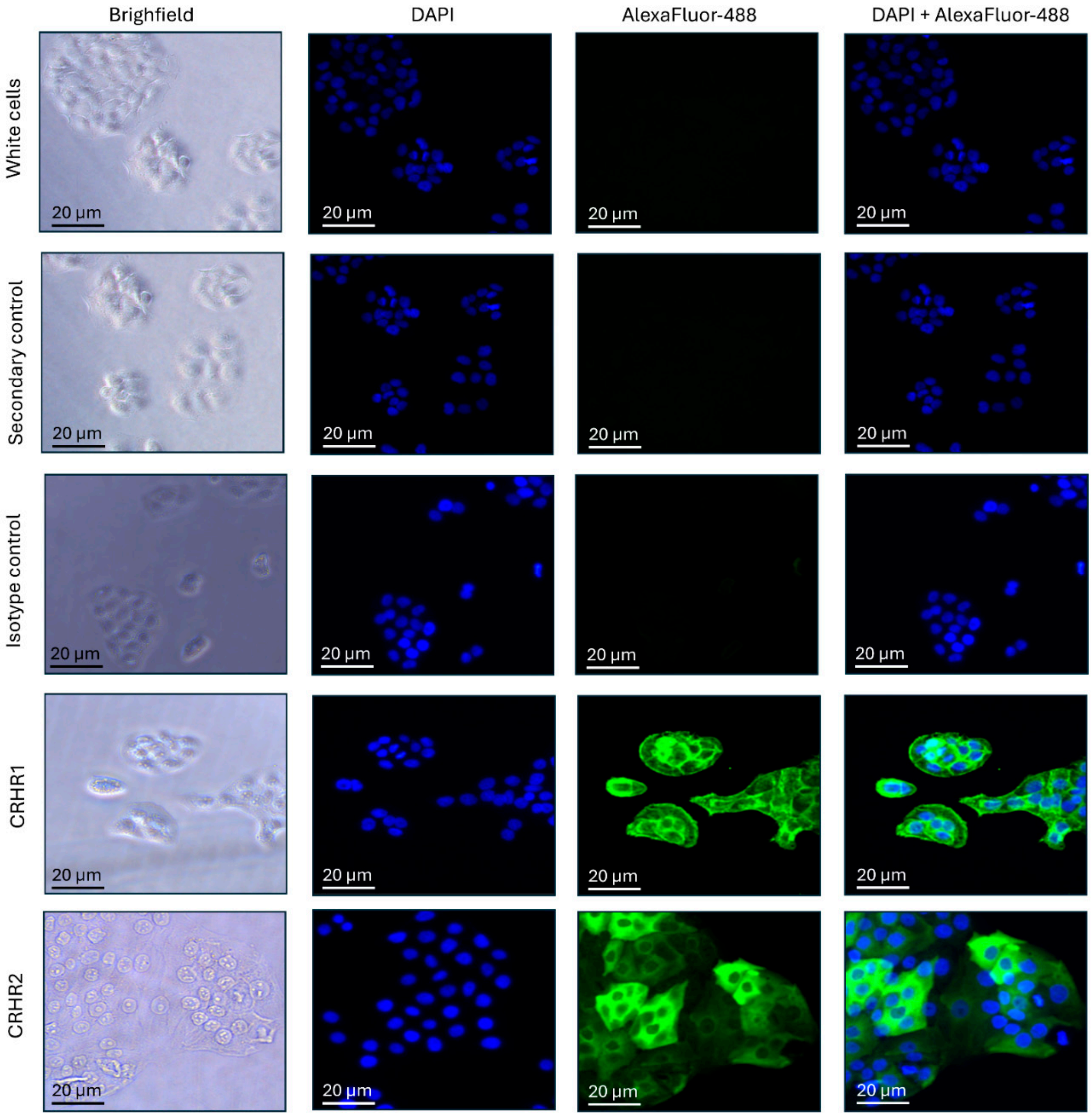

Figure S3: Fluorescent microscopy imaging of CRHR1/R2 receptors with immunolabeling controls. Labeling with specific anti-CRHR1/2 antibodies were performed on HaCaT cells in culture. CRHR1/2 were detected using an Alexa Fluor 488–conjugated secondary antibody (green). Nuclei were counterstained with DAPI (blue). Images were acquired with a 200x magnification.

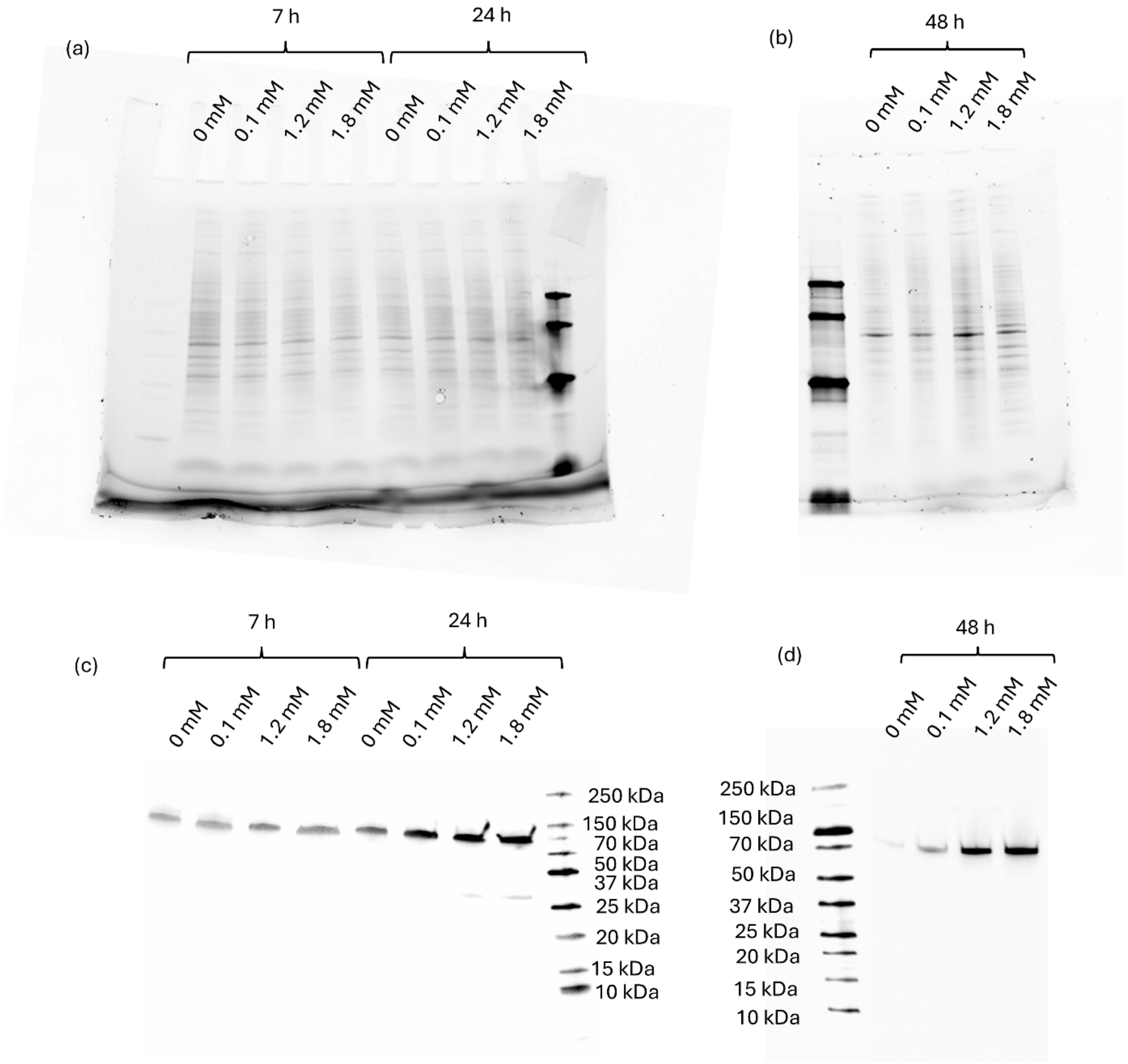

Figure S4: Pictures of stain-free gels and immunoblot membranes of involucrin during calcium-induced differentiation of HaCaT cells. Pictures (a) and (b) show total protein profiles revealed by the stain-free method. Chemiluminescence pictures (c) and (d) show representative membranes with specific revelation of involucrin

protein. Protein expression was analyzed in cell lysates incubated with 0, 0.1, 1.2, and 1.8 mM calcium for 7 and 24 hours (a and c) or 48 hours (b and d).

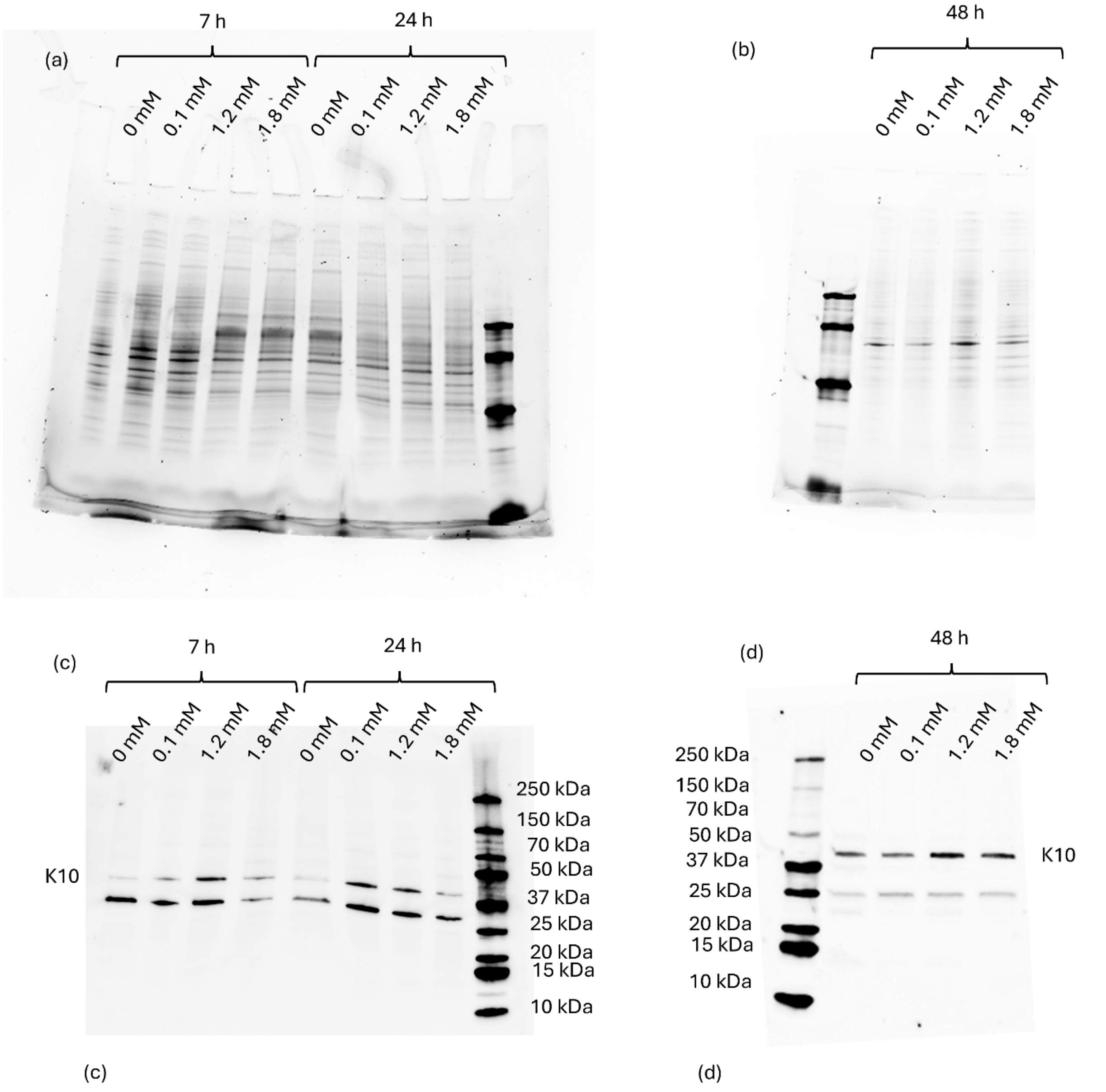

Figure S5: Pictures of stain-free gels and immunoblot membranes of cytokeratin K10 during calcium-induced differentiation of HaCaT cells. Pictures (a) and (b) show total protein profiles revealed by the stain-free method. Chemiluminescence pictures (c) and (d) show representative membranes with specific revelation of cytokeratin K10 protein. Protein expression was analyzed in cell lysates incubated with 0, 0.1, 1.2, and 1.8 mM calcium for 7 and 24 hours (a and c) or 48 hours (b and d).

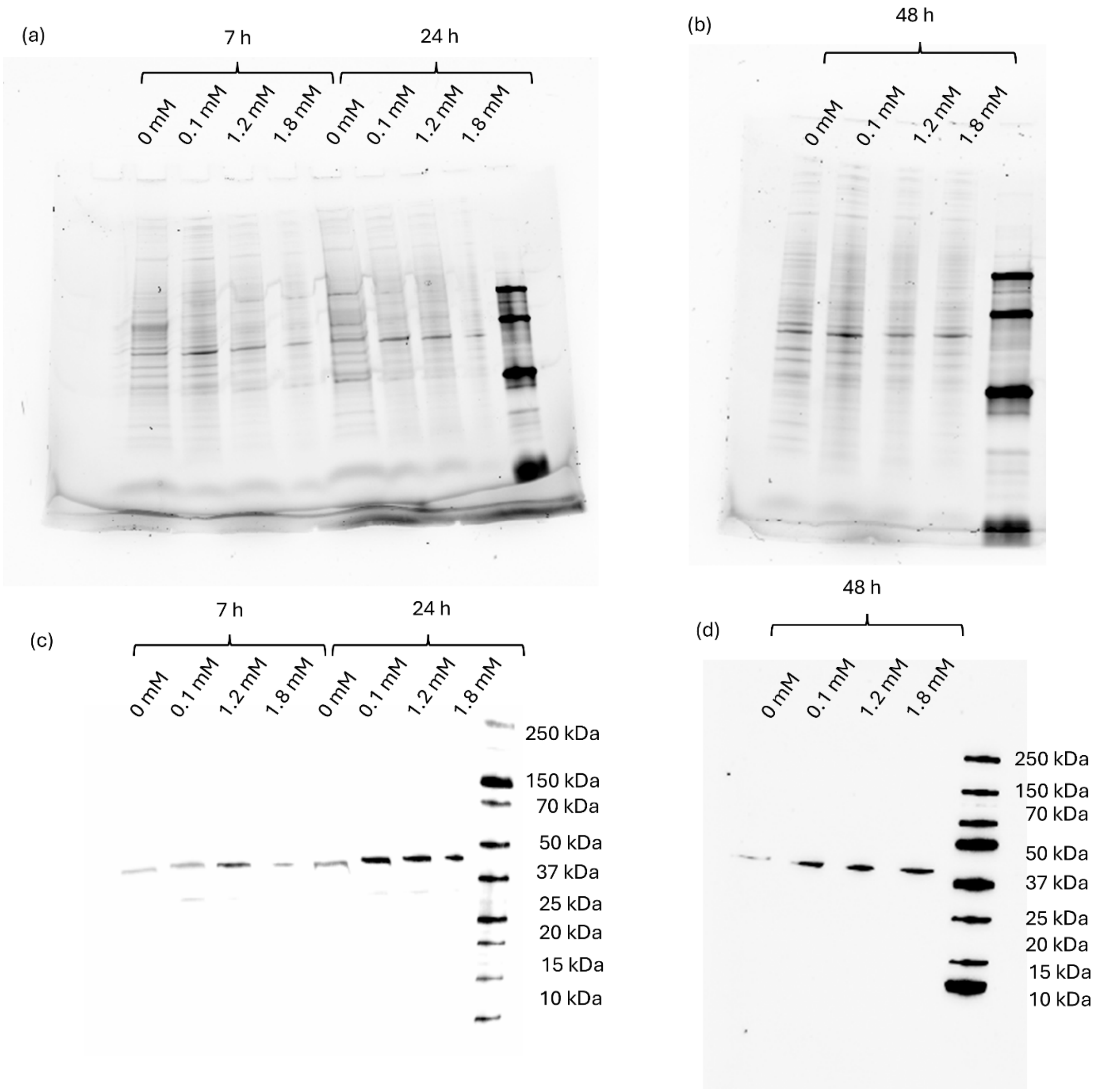

Figure S6: Pictures of stain-free gels and immunoblot membranes of CRHR1 during calcium-induced differentiation of HaCaT cells. Pictures (a) and (b) show total protein profiles revealed by the stain-free method. Chemiluminescence pictures (c) and (d) show representative membranes with specific revelation of CRHR1 protein. Protein expression was analyzed in cell lysates incubated with 0, 0.1, 1.2, and 1.8 mM calcium for 7 and 24 hours (a and c) or 48 hours (b and d).

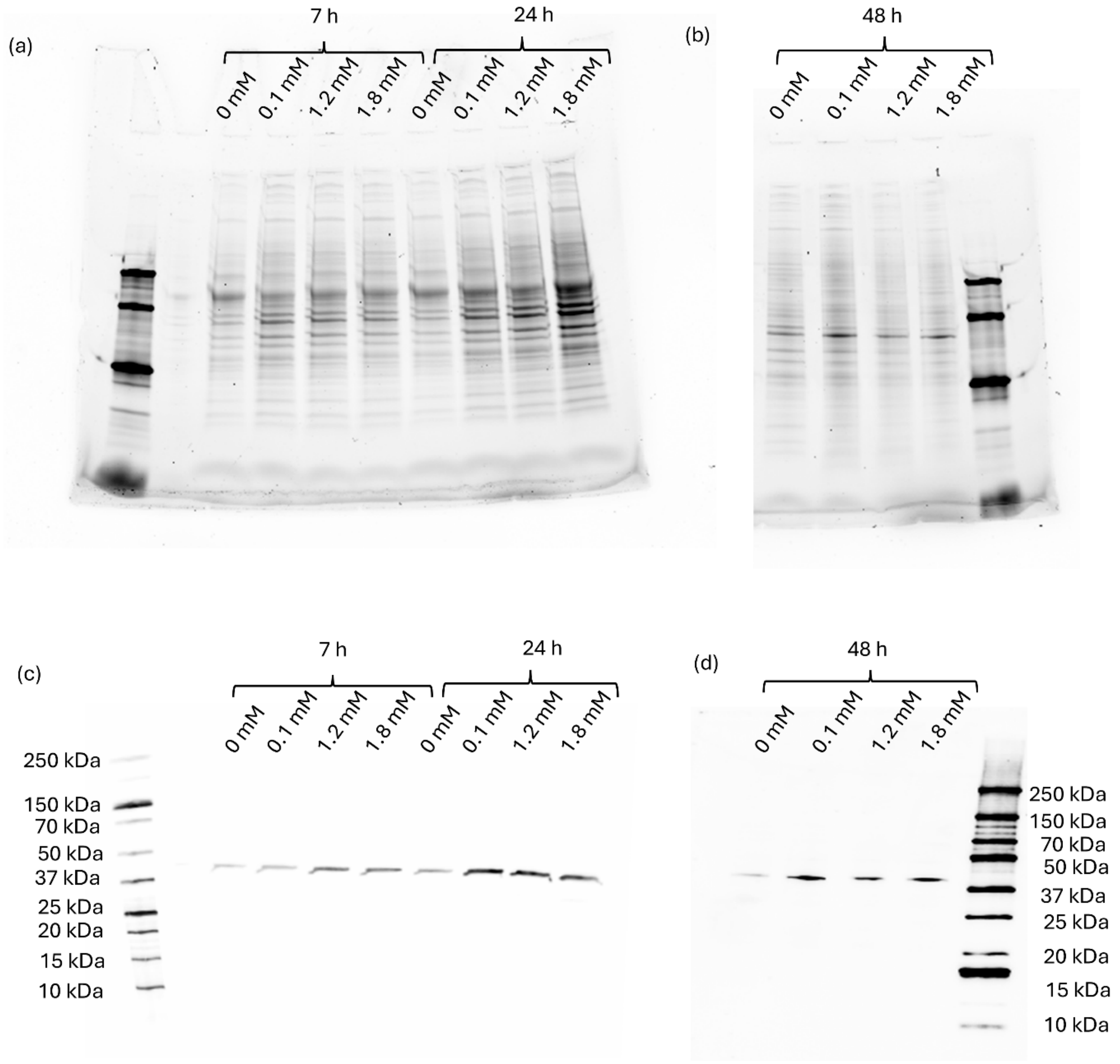

Figure S7: Pictures of stain-free gels and immunoblot membranes of CRHR2 during calcium-induced differentiation of HaCaT cells. Pictures (a) and (b) show total protein profiles revealed by the stain-free method. Chemiluminescence pictures (c) and (d) show representative membranes with specific revelation of CRHR2 protein. Protein expression was analyzed in cell lysates incubated with 0, 0.1, 1.2, and 1.8 mM calcium for 7 and 24 hours (a and c) or 48 hours (b and d).

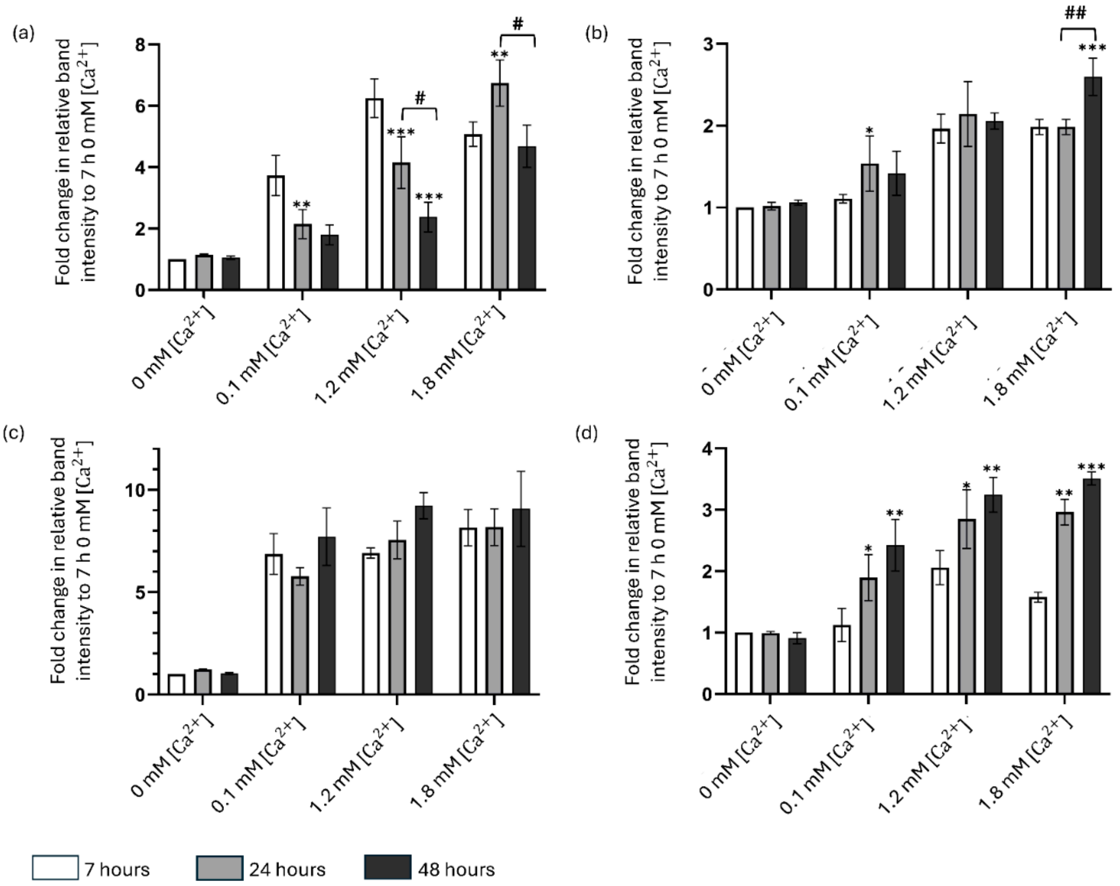

Figure S8: Western blot quantification of CRHR1/R2 proteins in HaCaT cells. Proteins expressions were studied at 7, 24 and 48 hours and at various calcium concentrations, respectively 0, 0.1, 1.2 and 1.8 mM. Graphs show quantifications of band for (a) involucrin, (b) cytokeratin K10, (c) CRHR1, and (d) CRHR2. Intensities are expressed as fold change using the 0 mM calcium concentration at 7 h as reference, Values are means ± SD, N = 5. # $p < 0.05$; ## $p < 0.01$; ### $p < 0.001$; #### $p < 0.0001$. Significative values in comparison with 7h means *$p < 0.05$; ** $p < 0.01$; *** $p < 0.001$; **** $p < 0.0001$

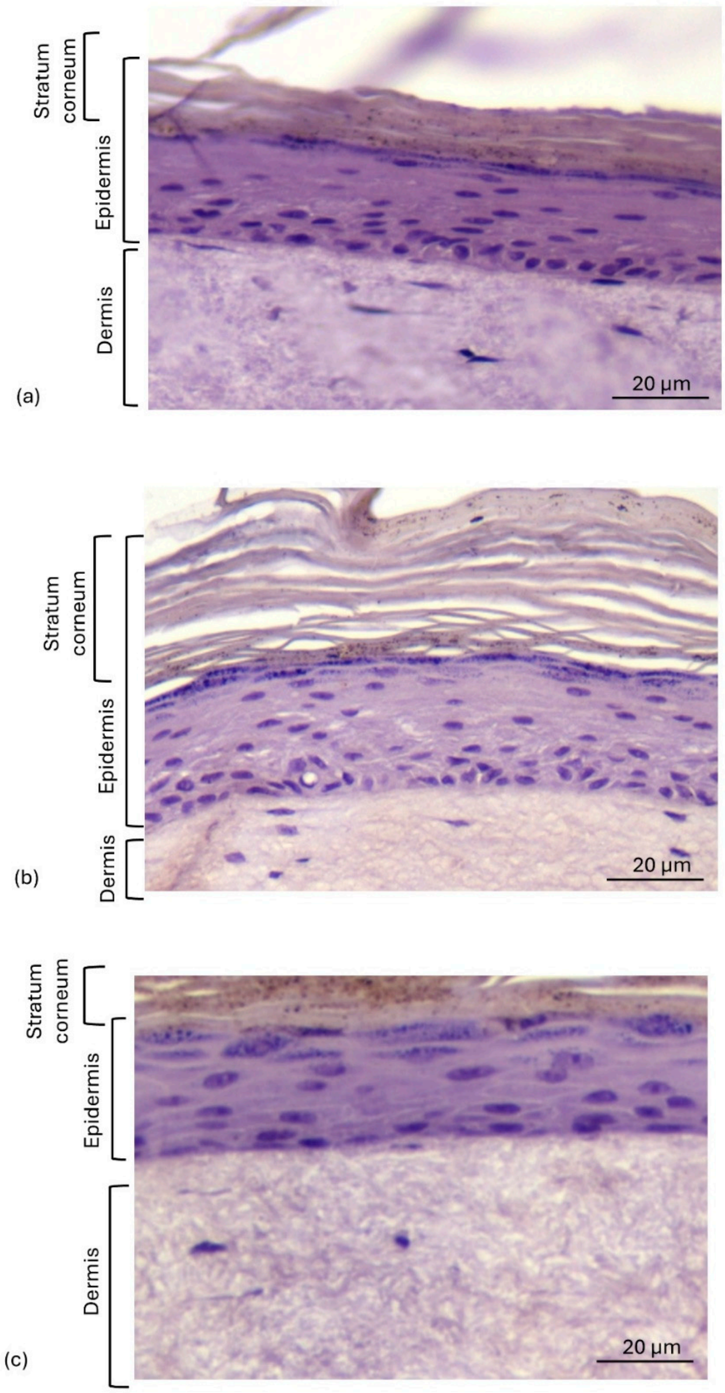

Figure S9: Experimental controls for immunolabeling of CRHR1 and CRHR2 proteins in human reconstructed skin. Signal, if any, would appears in brown on skin sections. Slides were counterstained with Mayer's haematoxylin. Three control conditions were performed with: no antibodies (a), with isotype control (b), and with secondary antibody only (c). Optical microscopy images were acquired with a 200× magnification. Representative pictures.
